# Usefulness of ECG criteria to rule out left ventricular hypertrophy in older individuals with true left bundle branch block: an observational study

**DOI:** 10.1186/s12872-021-02332-8

**Published:** 2021-11-17

**Authors:** Caio Assis Moura Tavares, Nelson Samesima, Felippe Lazar Neto, Ludhmila Abrahão Hajjar, Lucas C. Godoy, Eduardo Messias Hirano Padrão, Mirella Facin, Wilson Jacob Filho, Michael E. Farkouh, Carlos Alberto Pastore

**Affiliations:** 1 Instituto do Coracao (InCor), Hospital das Clinicas HCFMUSP, Faculdade de Medicina, Universidade de Sao Paulo, Sao Paulo, Brazil; 2 Peter Munk Cardiac Centre and Heart and Stroke/Richard Lewar Centre of Excellence in Cardiovascular Research, University of Toronto, Toronto, ON Canada; 3 Unidade de Eletrocardiografia, Instituto do Coracao, Hospital das Clínicas, Faculdade de Medicina, Universidade de Sao Paulo, Av. Dr Enéas de Carvalho Aguiar, 44, andar AB, Sao Paulo, SP 05403-900 Brazil

**Keywords:** Hypertension, Electrocardiography, Left bundle branch block, Elderly

## Abstract

**Background:**

Advanced age is associated with both left bundle branch block (LBBB) and hypertension and the usefulness of ECG criteria to detect left ventricular hypertrophy (LVH) in patients with LBBB is still unclear. The diagnostic performance and clinical applicability of ECG-based LVH criteria in patients with LBBB defined by stricter ECG criteria is unknown. The aim of this study was to compare diagnostic accuracy and clinical utility of ECG criteria in patients with advanced age and strict LBBB criteria.

**Methods:**

Retrospective single-center study conducted from Jan/2017 to Mar/2018. Patients undergoing both ECG and echocardiogram examinations were included. Ten criteria for ECG-based LVH were compared using LVH defined by the echocardiogram as the gold standard. Sensitivity, specificity, predictive values, likelihood ratios, AUC, and the Brier score were used to compare diagnostic performance and a decision curve analysis was performed.

**Results:**

From 4621 screened patients, 68 were included, median age was 78.4 years, (IQR 73.3–83.4), 73.5% with hypertension. All ECG criteria failed to provide accurate discrimination of LVH with AUC range between 0.54 and 0.67, and no ECG criteria had a balanced tradeoff between sensitivity and specificity. No ECG criteria consistently improved the net benefit compared to the strategy of performing routine echocardiogram in all patients in the decision curve analysis within the 10–60% probability threshold range.

**Conclusion:**

ECG-based criteria for LVH in patients with advanced age and true LBBB lack diagnostic accuracy or clinical usefulness and should not be routinely assessed.

**Supplementary Information:**

The online version contains supplementary material available at 10.1186/s12872-021-02332-8.

## Introduction

Left ventricular hypertrophy (LVH) is an ultimate consequence of long-standing hypertension and is associated with all-cause mortality [1]. In patients receiving anti-hypertensive therapy, the improvement of LVH, as evaluated by the electrocardiogram (ECG), is associated with improved cardiovascular prognosis [2, 3]. Accordingly, current clinical practice guidelines recommend using the ECG as part of the routine assessment of patients with hypertension at baseline and during follow-up [4].

Abnormal depolarization of the left ventricle due to left bundle branch block (LBBB) may compromise the electrocardiographic diagnose of LVH because the LVH diagnostic criteria were developed and validated in patients without conduction disturbances [5]. More recent studies that evaluated ECG diagnostic performance in patients with LBBB, were heterogeneous and had a wide range of sensitivities due to the inclusion of different populations, use of multiple cut-offs, and distinct criteria [6], creating barriers for implementation into clinical practice. In fact, some authors even consider that the electrocardiographic diagnosis of LVH should not be applied in those with LBBB [7]. The lack of universally accepted standards to distinguish true LBBB from conduction delay [8] adds even more complexity to this topic.

Advanced age is associated with hypertension and LBBB [9–11], and the number of patients with both conditions is expected to grow progressively in the next years because of the overall aging population. Validating or developing new accurate ECG criteria for LVH in this population can have relevant and immediate clinical applicability. We aim to evaluate the diagnostic accuracy of the traditional ECG-based LVH criteria in patients with LBBB and the clinical usefulness of using a selective strategy to guide echocardiogram orders.

## Methods

### Population

**Fig. 1:**
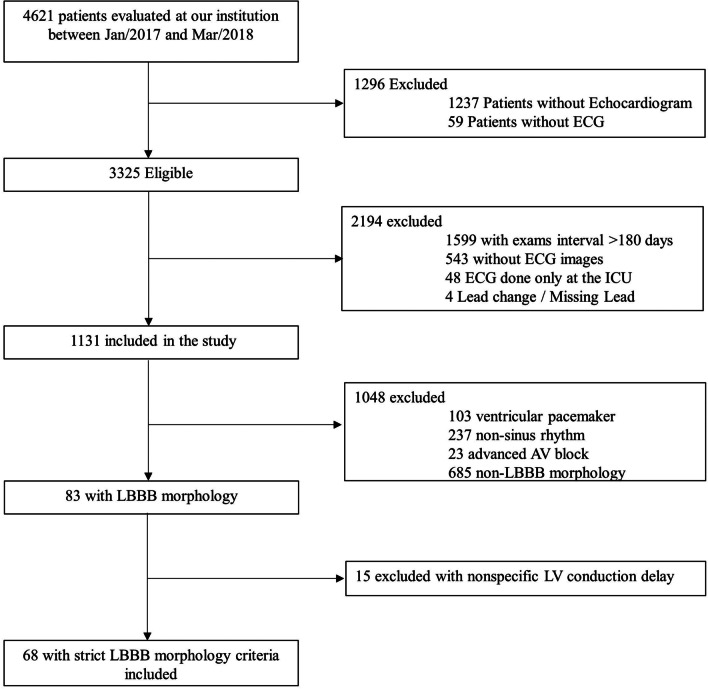
Study flowchart. AV = atrioventricular; ECG = electrocardiography; ICU = Intensive Care Unit; LBBB = Left Bundle Branch Block

We retrospectively collected data from patients ≥ 70 years old (as of March/31/2018) evaluated at a tertiary care teaching hospital in Sao Paulo, Brazil. From January/2017 to March/2018, all outpatients and inpatients in non-critical care units patients who underwent a 12-lead ECG and echocardiogram from January/2017 to March/2018 were deemed eligible. Exclusion criteria were time between ECG and echocardiogram greater than 180 days, ECG images that could not be retrieved, only ECG images available from the ICU and ECG with lead changes or missing leads. ECG tracings were then inspected to exclude patients with ventricular pacemaker, non-sinus rhythm, advanced atrioventricular block or QRS with non-LBBB morphology (Fig. 1).

### ECG analysis

Standard 12-lead ECGs were acquired at 10 mm/mV calibration and speed of 25 mm/s. Two physicians (CAMT and EMP) independently screened all tracings for LBBB, using the strict definition proposed by Strauss et al. (available in the Additional file 1: Table S1). As the LBBB identified by the ECG can represent conduction disease, left ventricle myocardial disease or a combination of both, the stricter LBBB criteria proposed by Strauss identify only those with true conduction disease and were developed aiming to predict who would better respond to cardiac resynchronization therapy (CRT) [12]. In cases of discordance between the examiners, the tracings were reviewed together with an experienced cardiologist (CAP) and the classification was defined based on consensus. Two cardiologists (NS and MF), blinded to echocardiogram and clinical data, calculated the following LVH criteria in all ECGs with LBBB: Peguero-Lo Presti, Cornell voltage, Cornell Voltage duration, SV2 plus SV3, R wave in avL, R wave product in avL, Sokolow-Lyon, Sokolow-Lyon product, Dalfó criteria and Gubner-Ungerleider (Additional file 1: Table S2). In case of discordances, a third cardiologist also revised the ECG tracings (CAMT).

### Echocardiographic analysis

Echocardiograms were used as the gold-standard method to diagnose LVH. All echocardiograms were performed at our institution according to international guidelines [12]. Left Ventricular Mass was calculated using the Devereux formula: left ventricular mass (g) = 0,80 × 1,04 [(septal thickness + internal diameter + posterior wall thickness)^3^ − (internal diameter)^3^] + 0.6 g[13], and indexed by the Body Surface Area (BSA), calculated by the Dubois Formula (BSA = 0.007184 × height (m)^0.725^ × weight (kg)^0.425^, with LVH defined as > 95 g/m^2^ in females and > 115 g/m^2^ male subjects.

### Clinical data

Epidemiological data from all patients were retrieved from the electronic medical record: anthropometric data (height, weight, body mass index), age in years (at the day of echocardiogram exam), comorbidities as diagnosed by the attending physician (hypertension, diabetes, coronary artery disease, prior myocardial infarction, coronary artery bypass graft surgery, prior percutaneous coronary intervention, atrial fibrillation, peripheral artery disease, chronic obstructive pulmonary disease), medications prescribed (beta-blockers, calcium channel blockers, diuretics, angiotensin-converting enzyme inhibitors (ACEi), angiotensin II receptors blockers (ARB), hydralazine/nitrate). Vital signs were obtained through chart review (blood pressure and heart rate).

### Statistical analysis

Clinical and echocardiographic characteristics were summarized as median and interquartile range or proportions, based on LVH status in the echocardiogram. The rank sum test was used for comparing continuous variables between groups and the Fisher exact test for categorical variables. For each ECG criterion, sensitivity, specificity, positive and negative predictive values, and likelihood ratios were calculated using the echocardiogram as the gold standard. Pearson correlation coefficients between ECG criteria and left ventricular mass index were calculated using the ECG criteria as continuous variables. Discriminative performance of the ECG criteria was calculated according to the area under the curve (AUC) of the receiver operating characteristic (ROC) curve. The Brier Score was calculated as a measure of overall performance, defined as the mean squared difference between the observed (Echo-LVH) and predicted outcome (ECG-LVH, for each ECG criteria). The Brier Score ranges from 0 to 1 with lower values being indicative of better overall performance.

We used a decision curve analysis framework to assess how each ECG criterion would impact clinical practice. In brief, the net-benefit for each ECG criterion was calculated by subtracting the proportion of false positives from the true positives, weighted by the relative harm of a false positive and a false negative result. Each score was then compared to strategies of ordering echocardiogram for all or none, by subtracting the estimated net-benefit of ordering-all strategy from the respective criteria. This method considers how many false positives cases the physician is willing to treat to avoid not treating a false negative patient. In our case, this can be translated as how many echocardiograms without LVH the physician is willing to accept to avoid not recognizing one echocardiogram with LVH, which could guide the physician’s decision on echocardiogram orders. The threshold probabilities were selected a priori with a large threshold range (0.1–0.6) to mirror different resource settings. Detailed explanation of the decision curve analysis can be found elsewhere [13]. Our manuscript was based in the 2015 Standards for Reporting Diagnostic Accuracy Studies (STARD), available in the Additional file 1: Table S3. A two-sided *p* < 0.05 was considered statistically significant. Statistical analysis was performed using the R software, version 3.6.2 (R Project for Statistical Computing).

## Results

### Characteristics of the study population

As outlined in Fig. 1, after screening 4621 patients, 68 patients with LBBB criteria were included, of whom 46 (67.6%) had LVH based on the echocardiogram. The median age was 78.4 years (IQR 73.3–83.4), most were female (n = 38, 55.9%), and hypertension was the most common chronic disease (n = 50, 73.5%); followed by coronary artery disease (n = 32, 47.1%) and dyslipidemia (n = 27, 39.7%). The median time interval between the ECG and the echocardiogram was 14 days (IQR 1.0–43.3). Patients with LVH were older and predominantly male. Demographic data of the population is summarized in Table 1. As expected, echocardiographic diagnosis of LVH was associated with distinct echocardiographic parameters: lower ejection fraction (46.5% versus 59.5%, *p* = 0.027), higher left ventricular mass index (141.0 versus 97.5 g/m^2^, *p* < 0.001), increased left atrium diameter (46.0 versus 38.0 mm, *p* < 0.001), left ventricular end-diastolic diameter (57.5 versus 48.0 mm, *p* = 0.001), and left ventricular end-systolic diameter (43.5 versus 34.0 mm, *p* = 0.004), as shown in Table 2.

**Table 1:** Demographic data

Demographic data	All patients (n = 68)	LVH patients (n = 46)	Non-LVH patients (n = 22)	*P* value
Age (years)	78.4 (73.3–83.4)	78.7 (74.5–79.9)	76.2 (71.6–80.6)	0.018
Female	38 (55.9%)	19 (41.3%)	19 (86.4%)	<0.001
BMI (kg/m^2^)	25.3 (23–8–27.8)	24.8 (22.9–27.7)	26.0 (24.2–27.7)	0.235
SBP (mmHg)	120.0 (110.0–140.0)	120.0 (110.0–132.0)	121.0 (112.5–140.0)	0.256
DBP (mmHg)	71.5(60.0–80.0)	70.0 (60.0–80.0)	80.0 (71.5–80.0)	0.033
Heart rate (bpm)	67.0 (57.0–77.0)	69.0 (55.5–77.8)	65.0 (59.0–73.8)	0.854
Hypertension	50 (73.5%)	33 (71.7%)	17 (77.3%)	0.772
Type 2 diabetes	19 (27.9%)	10 (21.7%)	9 (40.9%)	0.148
Dyslipidemia	27 (39.7%)	18 (39.1%)	9 (40.9%)	1.000
Paroxysmal atrial fibrillation	11 (16.2%)	9 (19.6%)	2 (9.1%)	0.482
Coronary artery disease	32 (47.1%)	19 (41.3%)	13 (59.1%)	0.201
Previous myocardial infarction	17 (25.0%)	11 (23.9%)	6 (27.3%)	0.772
Previous CABG	14 (20.6%)	7 (15.2%)	7 (31.8%)	0.198
Previous PCI	18 (26.5%)	12 (26.1%)	6 (27.3%)	1.000
Peripheral artery disease	6 (8.8%)	4 (8.7%)	2 (9.1%)	1.000
Chronic obstructive pulmonary disease	8 (11.8%)	6 (13.0%)	2 (9.1%)	1.000
*Medication use*				
ACEi	31 (45.6%)	20 (43.5%)	11 (50.0%)	0.795
ARBs	19 (27.9%)	13 (28.3%)	6 (27.3%)	1.000
CCBs	14 (20.6%)	8 (17.4%)	6 (27.3%)	0.356
Beta blocker	47 (69.1%)	33 (71.7%)	14 (63.76%)	0.579
Hydralazine/Nitrate	11 (16.2%)	8 (17.4%)	3 (13.6%)	1.000
Diuretic	44 (64.7%)	28 (60.9%)	16 (72.7%)	0.421
Days between echocardiogram and ECG	14 (1.0–43.3)	13.5(1.0–42.8)	14 (3.3–47.8)	0.506

Demographic data of the cohort, according to the left ventricular hypertrophy status evaluated by echocardiography. Values are median and interquartile range or n (%)

ACEi: = Angiotensin-Converting Enzyme inhibitors; ARBs = Angiotensin Receptor blockers; BMI = Body Mass Index; CABG = Coronary Artery Bypass Graft; CCBs = Calcium Channel Blockers; DBP = Diastolic Blood Pressure; PCI = Percutaneous Coronary Intervention; SBP = Systolic Blood Pressure

**Table 2:** Echocardiographic parameters

Echocardiographic parameters	LVH patients (n = 46)	Non-LVH patients (n = 22)	*P* value
Ejection fraction (%)	46.5 (30.0–60.8)	59.5 (40.3–65.8)	0.027
Left ventricular mass index (g/m^2^)	141.0 (117.8–173.5)	97.5 (86.5–108.5)	< 0.001
Relative wall thickness (no unit)	0.37 (0.30–0.44)	0.41 (0.33–0.45)	< 0.306
Left Atrium diameter (mm)	46.0 (42.0–49.0)	38.0 (36.0–43.0)	< 0.001
Interventricular septal diameter (mm)	10.5 (9.3–10.7)	10.0 (9.0–11.0)	0.085
Posterior wall diameter (mm)	10.0 (9.0–11.0)	10.0 (9.0–10.0)	0.088
Left ventricular end- diastolic diameter (mm)	57.5 (50.0–65.8)	48.0 (47.0–55.8)	0.001
Left ventricular end-systolic diameter (mm)	43.5 (33.8–55.5)	34.0 (31.3–37.8)	0.004
Moderate or severe aortic stenosis	9 (19.6%)	2 (9.1%)	0.482
Moderate or severe mitral regurgitation	11 (23.9%)	1 (4.5%)	0.086
Moderate or severe aortic regurgitation	4 (8.7%)	1 (4.5%)	1.000

Echocardiographic parameters of cohort, according to the left ventricular hypertrophy status evaluated by echocardiography. Values are median and interquartile range or n (%)

### Diagnostic performance of the ECG criteria

*Sensitivity, specificity, predictive values and likelihood ratios* The standard cut-offs of the ECG criteria had a wide range of sensitivities (26.1–100%) and specificities (0–81.8%) (Table 3). No single ECG criterion had a good balance for both indices. The Peguero-Lo Presti and the Cornell Voltage Duration Product criteria had high sensitivity, but low specificity (sensitivity 100%, specificity 9.1% and sensitivity 97.8%, specificity 27.3%, respectively), whereas the Sokolow-Lyon and R wave in lead avL criteria had high specificity but low sensitivity (specificity 81.8%, sensitivity 26.1% and specificity 81.8%, sensitivity 26.1%, respectively). Nominally, the highest positive likelihood ratio was observed for R avL (1.48) and the lowest negative likelihood ratio for Cornell Voltage Duration Product. Overall, the R avL voltage duration product had the highest positive predictive value (75.6%) and the Cornell Voltage Duration Product the highest negative predictive value (100%).

**Table 3:** Clinical utility of the ECG criteria

ECG criteria	Sensitivity (%) (95% CI)	Specificity (%) (95% CI)	PPV (%) (95% CI)	LR+ (95% CI)	NPV (%) (95% CI)	LR− (95% CI)
Peguero-Lo Presti	96.0 (88.6–100)	9.1 (0–22.7)	68.8 (57.2–79.0)	1.05 (0.93–1.24)	50.0 (0–100)	0.48 (0–1.86)
Cornell voltage	87.0 (76.0–96.1)	27.3 (8.0–47.4)	71.4 (59.3–83.7)	1.2 (0.92–1.67)	50.0 (22.2–78.6)	0.48 (0.1–1.46)
Cornell VDP	100 (NA)	9.1 (0–22.2)	69.7 (58.2–80.0)	1.10 (1.00–1.28)	100 (NA)	0 (NA)
SV2 + SV3	39.1 (26.1–53.8)	59.1 (36.9–79.2)	66.7 (50.0–84.4)	0.96 (0.50–2.15)	31.7 (17.5–46.5)	1.03 (0.70–1.70)
R aVL	26.1 (13.3–38.7)	81.8 (63.2–95.7)	75.0 (50.0–93.8)	0.96 (0.50–2.15)	34.6 (21.6–48.2)	0.90 (0.69–1.19)
R aVL VDP	67.4 (53.2–80.0)	54.6 (33.3–76.2)	75.6 (61.1–88.4)	1.48 (0.92–2.69)	44.4 (26.1–65.2)	0.60 (0.31–1.08)
Sokolow-Lyon	26.1 (13.7–41.3)	81.8 (63.0–95.7)	75.0 (50.1–94.7)	1.43 (0.53–6.13)	34.6 (22.2–47.9)	0.90 (0.70–1.23)
Sokolow-Lyon VDP	60.9 (46.5–73.5)	50.0 (29.2–71.4)	71.8 (56.4–85.4)	1.22 (0.73–2.18)	37.9 (20.7–54.6)	0.78 (0.46–1.40)
Gubner-Ungerleider	82.6 (70.6–93.2)	18.2 (63.2–95.7)	67.9 (55.8–79.3	1.01 (0.79–1.32)	33.3 (6.7–62.5)	0.96 (0.32–4.24)
Dalfó	95.7 (88.9–100)	0 (NA)	66.7 (55.9–78.5)	0.96 (0.89–1.00)	NA	NA

Diagnostic performance of all the ECG criteria in patients with left bundle branch block, using the echocardiogram based left ventricular hypertrophy as the gold-standard

CI = Confidence Interval; LR+ = positive likelihood ration; LR− = negative likelihood ratio; NA = non applicable; NPV = Negative Predictive Value; PPV = Positive Predictive Value; R aVL = R wave in lead aVL; SV2 + SV3 = sum of the S wave in V2 plus V3; VDP = voltage-duration product

*Discriminative power assessed by the area under the curve (AUC) and overall performance according to the Brier score* Discrimination of all ECG criteria was poor. The AUC ranged between 0.53 and 0.67 and the Brier score from 0.20 to 0.22. The AUC of the Cornell Voltage duration product was numerically higher than the other criteria (0.67, 95% CI 0.53–0.79) (Table 4).

**Table 4:** Area under the receiver operating characteristic curve (AUC) and brier score for all ECG criteria

ECG criteria	AUC (95% CI)	Brier score
Peguero-Lo Presti	0.59 (0.45–0.72)	0.21
Cornell Voltage	0.55 (0.40–0.68)	0.22
Cornell VDP	0.67 (0.53–0.79)	0.20
SV2 + SV3	0.53 (0.38–0.68)	0.22
R aVL	0.62 (0.46–0.76)	0.21
R aVL VDP	0.64 (0.49–0.78)	0.21
Sokolow-Lyon	0.54 (0.39–0.69)	0.22
Sokolow-Lyon VDP	0.57 (0.42–0.72)	0.22
Gubner-Ungerleider	0.54 (0.42–0.72)	0.22
Dalfó	0.55 (0.39–0.69)	0.22

AUC and Brier Score for the ECG criteria using the echocardiogram based left ventricular hypertrophy as the gold-standard

CI = Confidence Interval; R aVL = R wave in lead aVL; SV2 + SV3 = sum of the S wave in V2 plus V3; VDP = voltage-duration product

**Fig. 2:**
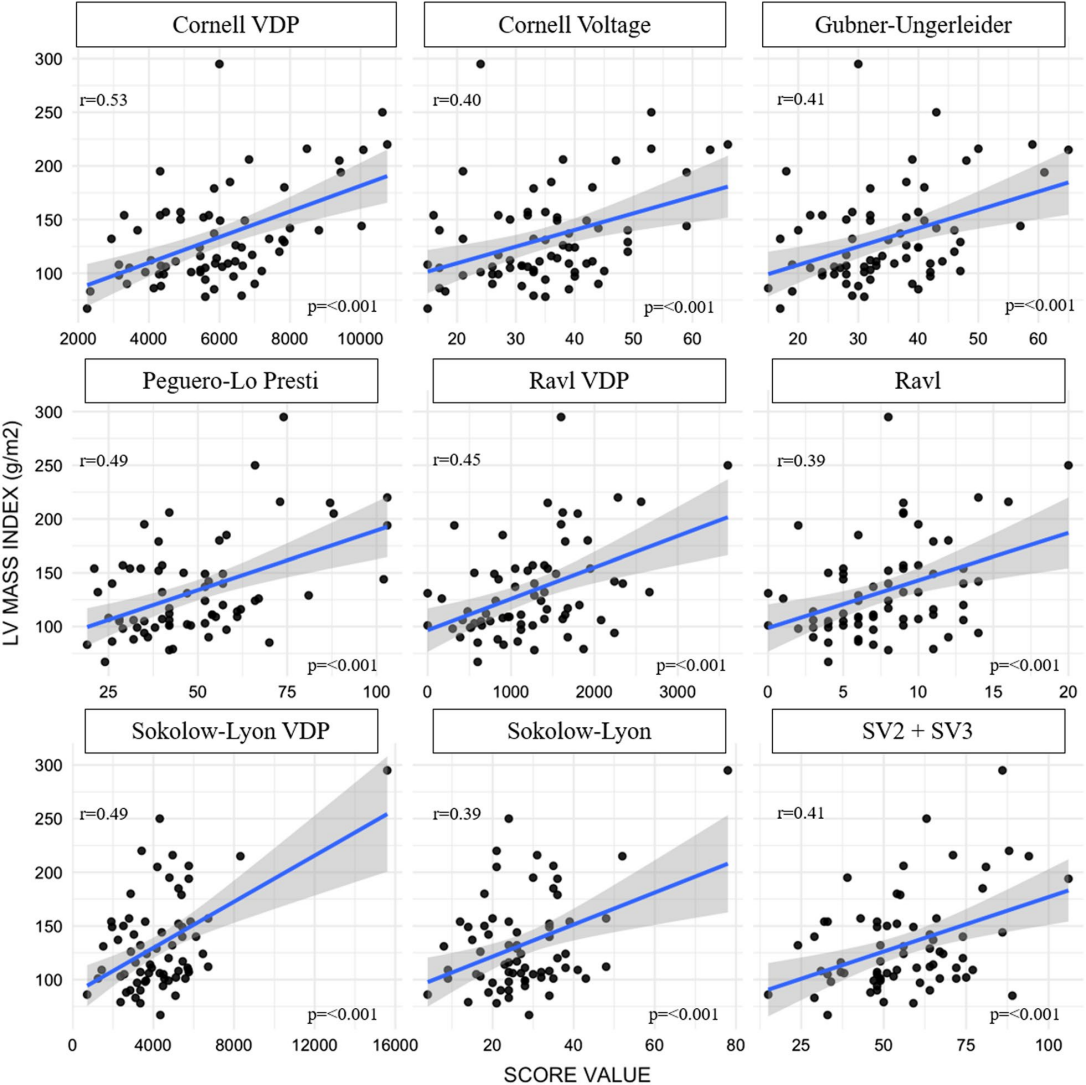
Correlation between ECG criteria and left ventricular mass index. LV = left ventricular; R aVL = R wave in lead aVL; SV2 + SV3 = sum of the S wave in V2 plus V3; VDP = voltage-duration product

*Correlation between ECG criteria and left ventricular mass index* Moderate correlation (0.39–0.53) was observed for all ECG criteria. Figure 2 summarizes the correlation coefficients between the left ventricular mass index and ECG criteria.

**Fig. 3:**
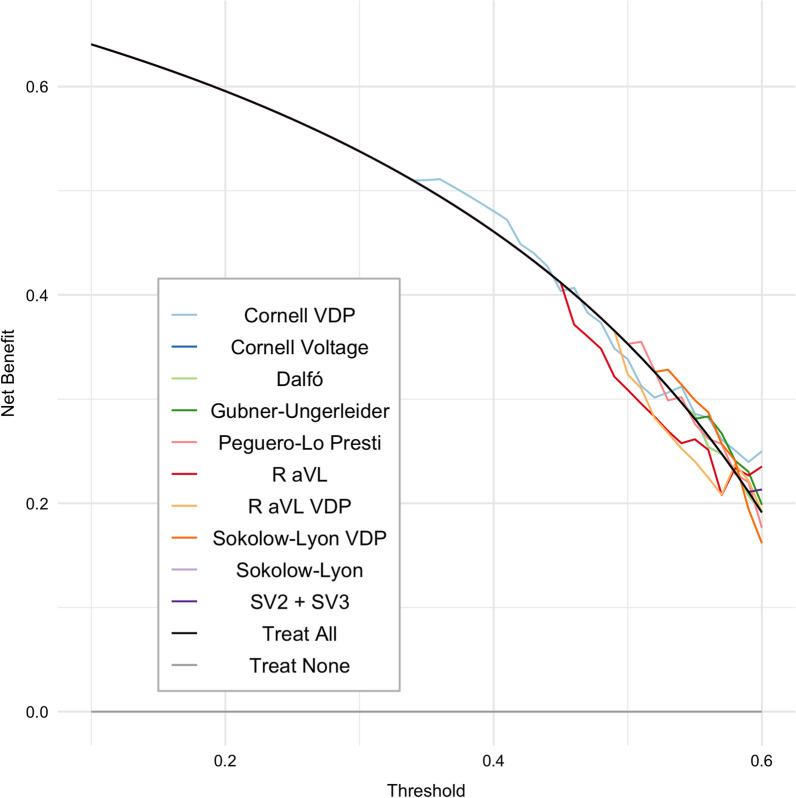
Decision curve analysis for the ECG criteria. Decision curve analysis of the ECG criteria for the detection of left ventricular hypertrophy as assessed by the echocardiogram. For different risk thresholds for which the clinician would opt to order an echocardiogram, the net benefit of each ECG criteria is plotted compared against strategies of echocardiogram to all patients (black line) or none (grey line). CI = Confidence Interval; R aVL = R wave in lead aVL; SV2 + SV3 = sum of the S wave in V2 plus V3; VDP = voltage-duration product

*Decision curve analysis* For all the tested threshold probabilities (10–60%) we found little to no clinical benefit of utilizing ECG criteria compared to a strategy of ordering echocardiograms for all patients (Fig. 3).

## Discussion

Our study had three main findings: first, the poor diagnostic performance of traditional ECG criteria in patients with strict criteria for LBBB; second, the low diagnostic accuracy of the recently proposed Peguero-Lo Presti criteria in patients with LBBB and third, the lack of clinical usefulness of the ECG criteria as a screening method for deciding on the need for an echocardiogram.

As expected, LBBB poises a challenging issue for ECG screening tests because it is associated with a high prevalence of LVH (over 40% in the literature [14–16] and 68% in our cohort). High pre-test probability of disease consequently demands a criterion to have an exceptionally low negative likelihood ratio to exclude the diagnosis. For instance, even though the Cornell Voltage Duration Product captured all patients with LVH, with a sensitivity of 100%, specificity was very low (9.1%), nearly classifying every patient as with ECG-based LVH, thus not useful as tool to discriminate/identify those with LVH. None of the other ECG scores and respective cut-offs tested had a negative predictive value (NPV) over 80% and therefore were unable to adequately rule-out LVH. One could alter criteria cut-offs; however, the analysis of the decision curve showed little to no clinical benefit in using these scores even when accounting for a wide range of thresholds.

These results question the usefulness of the LVH electrocardiographic criteria in patient with LBBB. Although some reports have yielded high sensitivity [17–21], especially when using a computer-assisted diagnostic system [6], other groups have also questioned the use of ECG-based LVH criteria in patients with LBBB [7]. Previous studies testing ECG-based LVH criteria in patients with LBBB were performed prior to the development of the stricter LBBB criteria, and likely enrolled a more heterogeneous population, mixing conduction disease with LVH or LV dilation. This limitation could have artificially improved the LVH diagnostic performance of the ECG.

Our highly selective approach identified a more homogenous group of patients with true conduction disease where ECG-based criteria were unsuitable to diagnose echocardiographic LVH. Because conventional LBBB criteria on the ECG represents a complex interplay between conduction system and LV muscle disease, applying ECG criteria to recognize mainly muscle disease (LVH) in those with established conduction disease (strict LBBB criteria) might lead to many false positives—as observed by the low positive likelihood ratios found in our study. Indeed, our results from the correlation coefficients between the ECG criteria and left ventricular mass support this hypothesis as well, where correlation coefficients were consistently weak/moderate for all studied ECG criteria.

Our findings also inform practice. The 2018 AHA/ACCF/HRS guidelines recommend that patients diagnosed with new LBBB should undergo screening with echocardiogram [22]. However, the approach for patients at risk for LVH and a baseline echocardiogram without LVH is still uncertain. Because of the long waiting time for echocardiogram in low- and middle-income countries [23], alternatives for LVH assessment are needed, as LVH might influence treatment decisions [24]. ECGs can be performed at a low cost and repeated on a regular basis [23], helping clinicians to identify patients who have evolving changes and are at high risk of adverse outcomes earlier. This approach can shorten echocardiogram waiting times for those deemed high risk and create an earlier therapeutic window for intervention. Nevertheless, based on our findings, the ECG criteria we tested are not reliable to guide selective screening of patients for LVH when LBBB is present, and, therefore, routine assessment of LVH based on ECG criteria should not be performed.

### Study limitations

This was a retrospective single-center study with inherent limitations that warrant acknowledgment. First, despite screening more than 4,000 patients, our final sample size is small, which mirrors the prevalence of LBBB. Second, we excluded patients with atrial fibrillation and other non-sinus rhythms to minimize differences between QRS voltage measurements and caution is needed when attempting to extrapolate our findings to these patients. Third, as our study was performed using a single ECG analysis, we have not evaluated if evolving changes in ECG criteria over time might have a role in the diagnosis or follow-up of ECG-based LVH. Fourth, our study did not address if ECG-based LVH (named electrical LVH) might provide prognostic information even in the absence of LVH as assessed by echocardiogram (named anatomic LVH) in patients with LBBB, as long-term outcomes were not available [25]. Finally, our population is representative of a tertiary cardiovascular reference center, where there is a high burden of cardiovascular disease, and the generalization of our findings to primary and secondary care settings may be limited.

## Conclusion

Our findings suggest no role for routine use of traditional LVH electrocardiographic criteria in patients with LBBB, neither for screening of LVH nor for guiding a selective approach to ordering echocardiograms.

## Supplementary Information

**Additional file 1**. Supplementary Tables S1–S3.

## Data Availability

The datasets generated and analyzed during the current study are available from the corresponding author on reasonable request.
