# Supplementary material for: Usefulness of ECG criteria to rule out left ventricular hypertrophy in older individuals with true left bundle branch block: an observational study
Source: BMC Cardiovasc Disord. 2021 Nov 17;21:547. doi: 10.1186/s12872-021-02332-8 (PMC8600759; doi:10.1186/s12872-021-02332-8)
Supplement: Supplementary file 1 — Additional file 1. Supplementary Tables S1–S3. [file 12872_2021_2332_MOESM1_ESM.docx]

**Additional file 1**

1. Additional file 1: Table S1
2. Additional file 1: Table S2
3. Additional file 1: Table S3
4. References

**Additional file 1: Table S1**. ECG criteria for LBBB as proposed by Strauss et al(1) and updated in 2018(2).

| **ECG criteria** | **Description** |
| --- | --- |
| QRS duration | ≥140 msec for males  ≥130 msec for females |
| Morphologic criteria for right-sided leads (V1, V2) | QS or rS in V1 and V2 |
| Mid-QRS notching or slurring^a^ | ≥2 leads including I, avL, V1, V2, V5 or V6 |

ECG features of stricter left bundle branch block criteria as proposed by Strauss. LBBB is diagnosed when all three criteria are fulfilled. ^a^ = defined as notches or slurs that starts 40 msec after the beginning of the QRS complex and before 50% of the QRS width.

**Additional file 1: Table S2**. ECG criteria and cut-offs for left ventricular hypertrophy in patients with Left Bundle Branch Block

| Criteria | Formula | Threshold | Reference |
| --- | --- | --- | --- |
| Peguero-Lo Presti | Deepest S wave + S _V4_ | ≥2.8mV males  ≥2.3mV females | (3) |
| Cornell Voltage | R_avL_ + S_V3_ | ≥2.8mV males  ≥2.0mV females | (4) |
| Cornell Voltage duration product | Males: (R_aVL_+S_V3_) *QRS duration  Females: (R_aVL_+S_V3_+0.6 mV) *QRS duration | >244 mV * msec | (5) |
| SV2 + SV3 | S_V2_ + S_V3_ | >6.0mV | (6) |
| R aVL | R_aVL_ | ≥1.1mV | (7) |
| R aVL duration product | R_aVL_*QRS duration | >103 mV * msec | (8) |
| Sokolow-Lyon | S_V1_ + Tallest R wave (V5 or V6) | ≥3.5mV | (7) |
| Sokolow-Lyon product | S_V1_ + Tallest R wave (V5 or V6)*QRS duration | >367.4 mV* msec males  >322.4 mV *msec females | (5) |
| Gubner-Ungerleider | R_I_ + S_III_ | >2.5mV | (9) |
| Dalfó | R_aVL_ + S_V3_ | >1.6mV males  > 1.4mV females | (10) |

**Additional file 1: Table S3**. The STARD 2015 guidelines checklist

|  | **Section & Topic** | **No** | **Item** | **Reported on page #** |
| --- | --- | --- | --- | --- |
|  |  |  |  |  |
|  | **TITLE OR ABSTRACT** |  |  |  |
|  |  | **1** | Identification as a study of diagnostic accuracy using at least one measure of accuracy  (such as sensitivity, specificity, predictive values, or AUC) | N/A |
|  | **ABSTRACT** |  |  |  |
|  |  | **2** | Structured summary of study design, methods, results, and conclusions  (for specific guidance, see STARD for Abstracts) | 2 |
|  | **INTRODUCTION** |  |  |  |
|  |  | **3** | Scientific and clinical background, including the intended use and clinical role of the index test | 4 |
|  |  | **4** | Study objectives and hypotheses | 4 |
|  | **METHODS** |  |  |  |
|  | *Study design* | **5** | Whether data collection was planned before the index test and reference standard  were performed (prospective study) or after (retrospective study) | 5 |
|  | *Participants* | **6** | Eligibility criteria | Figure 1 / page 5 |
|  |  | **7** | On what basis potentially eligible participants were identified  (such as symptoms, results from previous tests, inclusion in registry) | Figure 1 / page 5 |
|  |  | **8** | Where and when potentially eligible participants were identified (setting, location and dates) | Figure 1 / page 5 |
|  |  | **9** | Whether participants formed a consecutive, random or convenience series | Figure 1 / page 5 |
|  | *Test methods* | **10a** | Index test, in sufficient detail to allow replication | 6 |
|  |  | **10b** | Reference standard, in sufficient detail to allow replication | 6 |
|  |  | **11** | Rationale for choosing the reference standard (if alternatives exist) | 6 |
|  |  | **12a** | Definition of and rationale for test positivity cut-offs or result categories  of the index test, distinguishing pre-specified from exploratory | Supplementary table S2 |
|  |  | **12b** | Definition of and rationale for test positivity cut-offs or result categories  of the reference standard, distinguishing pre-specified from exploratory | Supplementary table S2 |
|  |  | **13a** | Whether clinical information and reference standard results were available  to the performers/readers of the index test | 5 |
|  |  | **13b** | Whether clinical information and index test results were available  to the assessors of the reference standard | 5 |
|  | *Analysis* | **14** | Methods for estimating or comparing measures of diagnostic accuracy | 6 and 7 |

|  | **Section & Topic** | **No** | **Item** | **Reported on page #** |
| --- | --- | --- | --- | --- |
|  |  | **15** | How indeterminate index test or reference standard results were handled | N/A |
|  |  | **16** | How missing data on the index test and reference standard were handled | N/A |
|  |  | **17** | Any analyses of variability in diagnostic accuracy, distinguishing pre-specified from exploratory | N/A |
|  |  | **18** | Intended sample size and how it was determined | N/A |
|  | **RESULTS** |  |  |  |
|  | *Participants* | **19** | Flow of participants, using a diagram | Figure 1 |
|  |  | **20** | Baseline demographic and clinical characteristics of participants | Table 1 |
|  |  | **21a** | Distribution of severity of disease in those with the target condition | Tables 1 and 2 |
|  |  | **21b** | Distribution of alternative diagnoses in those without the target condition | N/A |
|  |  | **22** | Time interval and any clinical interventions between index test and reference standard | Table 1 |
|  | *Test results* | **23** | Cross tabulation of the index test results (or their distribution)  by the results of the reference standard | Tables 3 and 4 |
|  |  | **24** | Estimates of diagnostic accuracy and their precision (such as 95% confidence intervals) | Tables 3 and 4 |
|  |  | **25** | Any adverse events from performing the index test or the reference standard | N/A |
|  | **DISCUSSION** |  |  |  |
|  |  | **26** | Study limitations, including sources of potential bias, statistical uncertainty, and generalisability | 11 |
|  |  | **27** | Implications for practice, including the intended use and clinical role of the index test | 11 |
|  | **OTHER INFORMATION** |  |  |  |
|  |  | **28** | Registration number and name of registry | N/A |
|  |  | **29** | Where the full study protocol can be accessed | N/A |
|  |  | **30** | Sources of funding and other support; role of funders | 11 |
|  |  |  |  |  |

**Abbreviations**: **N/A** = not applicable

**Additional file 1: References**

1. Strauss DG, Selvester RH, Wagner GS. Defining left bundle branch block in the era of cardiac resynchronization therapy. Am J Cardiol. 2011;107(6):927-34.

2. Zusterzeel R, Vicente J, Ochoa-Jimenez R, Zhu J, Couderc JP, Akinnagbe-Zusterzeel E, et al. The 43rd International Society for Computerized Electrocardiology ECG initiative for the automated detection of strict left bundle branch block. J Electrocardiol. 2018;51(6S):S25-S30.

3. Peguero JG, Lo Presti S, Perez J, Issa O, Brenes JC, Tolentino A. Electrocardiographic Criteria for the Diagnosis of Left Ventricular Hypertrophy. J Am Coll Cardiol. 2017;69(13):1694-703.

4. Casale PN, Devereux RB, Alonso DR, Campo E, Kligfield P. Improved sex-specific criteria of left ventricular hypertrophy for clinical and computer interpretation of electrocardiograms: validation with autopsy findings. Circulation. 1987;75(3):565-72.

5. Okin PM, Roman MJ, Devereux RB, Kligfield P. Electrocardiographic identification of increased left ventricular mass by simple voltage-duration products. J Am Coll Cardiol. 1995;25(2):417-23.

6. Baranowski R, Malek L, Prokopowicz D, Spiewak M, Misko J. Electrocardiographic diagnosis of the left ventricular hypertrophy in patients with left bundle branch block: is it necessary to verify old criteria? Cardiol J. 2012;19(6):591-6.

7. Sokolow M, Lyon TP. The ventricular complex in left ventricular hypertrophy as obtained by unipolar precordial and limb leads. Am Heart J. 1949;37(2):161-86.

8. ed CTn. Electrocardiography in clinical practice: Orlando: Grune-

Stratton

1986.

9. Gubner R, Ungerleider H. Electrocardiographic criteria of left ventricular hypertrophy: factors determinin the evolution of the electrocardiographic patterns in hypertrophy and bundle branch block. Archives of Internal Medicine. 1943;72(2):196-209.

10. Dalfó A, López-Contreras J, Gil M, Martín M, Bayó J, Vila MA, et al. L32: Electrocardiographic diagnostic of left ventricular hypertrophy(LVH). Proposal of modification of cornell criteria. American Journal of Hypertension. 1997;10(S2):206A-A.
